# Application of PainDETECT in pediatric chronic pain: how well does it identify neuropathic pain and its characteristics?

**DOI:** 10.1097/PR9.0000000000001109

**Published:** 2023-11-28

**Authors:** Courtney W. Hess, Amanda R. Van Orden, Giulia Mesaroli, Jennifer N. Stinson, David Borsook, Laura E. Simons

**Affiliations:** a Stanford University School of Medicine, Palo Alto, CA, USA; b The Hospital for Sick Children, Toronto, ON, Canada; c University of Toronto, Toronto, ON, Canada; d Mass General Hospital, Boston, MA, USA

**Keywords:** Neuropathic pain, Pediatrics, Chronic pain, Screening tool, Mechanical allodynia, Mechanical hyperalgesia

## Abstract

**Introduction:**

Neuropathic pain (NP) arises from nerve damage or disease, and when not defined, it can impair function and quality of life. Early detection allows for interventions that can enhance outcomes. Diagnosis of NP can be difficult if not properly evaluated. PainDETECT is a NP screening tool developed and successfully used in adults.

**Objectives:**

We evaluated the validity of painDETECT in a pediatric population.

**Methods:**

Adolescents and young adults (10–19 years old) completed painDETECT and quantitative sensory testing (QST), which assessed mechanical allodynia and hyperalgesia, common symptoms of NP. Pain diagnoses, including neuropathic pain (n = 10), were collected through documentation in the medical chart. Descriptive statistics were used to examine age, gender, pain diagnoses, and painDETECT scores. Kruskal–Wallis H tests were conducted to examine differences in QST results across painDETECT categorizations.

**Results:**

Youth with chronic pain (N = 110, M_age_ = 15.08 ± 2.4 years, N_female_ = 88) and peers without pain (N = 55, M_age_ = 15.84 ± 3.9 years, N_female_ = 39) completed the painDETECT. The painDETECT scores for youth with pain (M = 12.7 ± 6.76) were significantly higher than those for peers without pain (M = 2.05 ± 2.41). PainDETECT demonstrated 80% sensitivity and 33% specificity in a pediatric population. Individuals who screened positively on the PainDETECT had significantly higher mechanical allodynia (M = 0.640 ± 0.994) compared with those who screened negatively (M = 0.186 ± 0.499; *P* = 0.016).

**Conclusion:**

PainDETECT demonstrated the ability to screen for NP, and QST mechanical allodynia results were consistent with a positive NP screen. Results of the study offer preliminary support for the ongoing assessment of the painDETECT as a brief, inexpensive, and simple-to-use screening tool for pediatric patients with primary pain complaints.

## Abstract 2

Supplemental Digital Content is Available in the Text.

PainDETECT is a neuropathic pain screening tool that demonstrated preliminary evidence of its sensitivity to neuropathic pain features among youth with chronic pain, and warrants further evaluation.

## 1. Introduction

Neuropathic pain (NP) is a consequence of damage or disease affecting the somatosensory (peripheral or central) nervous system (SNS)^12,22,26,27^ and is most often chronic in nature.^18^ Symptoms of NP often include loss of sensation, tingling, shooting, or burning pain that may be spontaneous or evoked (eg, exacerbated responses to innocuous [mechanical allodynia] or painful [mechanical hyperalgesia] stimuli).^1,2,10,18,27^ Specific prevalence is unknown due to diagnostic difficulties^14^; however, researchers have estimated that 3% to 10% of the population experiences chronic NP.^18,20,25^

As with other chronic pain conditions, NP is associated with decrements in physical functioning, mental health, quality of life, and sleep efficiency and increased home and school stress.^14,22,27^ Delayed treatment can compound these effects.^6,16^ Although interdisciplinary pain treatment is considered the gold standard for youth with NP, gaining access to these programs can be challenging,^16^ and diagnostic ambiguity can further impede access.^4,15^

However, in pediatric populations, NP can be more challenging to diagnose^9,14,27^ if not evaluated by a specialist.^14–16^ Neuropathic pain in youth as in adults is idiosyncratic with variability in onset/development of NP across patients despite similar injuries^9^ and documented delayed NP presentation after injury.^27^ Moreover, definitions of what is considered NP at clinical presentation have shifted over time. Most recently, the definition of NP was updated and no longer includes *dysfunction* of the SNS, propelling complex regional pain syndrome (CRPS) to no longer be considered NP but rather a type of nociplastic pain.^12,26^

Therefore, although it is clear that NP can strongly benefit from early identification^12,14^ and specifically tailored treatment, barriers exist to receiving such care. Diagnosis of NP often requires specialized evaluations by pain providers and thus requires appropriate and timely referrals. Unfortunately, approximately 27% of patients seen in tertiary pain clinics arrive with undiagnosed NP,^20,25^ despite patients visiting an average of 4 providers before their pain evaluation.^4^ Furthermore, patients report waiting, on average, 197.5 days for their pain clinic visit.^16^ Taken together, youth with chronic pain, including NP, experience substantial delays in their diagnosis and thus access or initiation of appropriate treatment.

To improve the referral process and therefore timely diagnosis and access to appropriate treatment recommendations,^21^ development and implementation of an NP screening tool is needed. Screening tools can begin the process of asserting disease of the SNS^14,18,27^ and differentiating NP from other types of pain—including nociceptive and musculoskeletal pain—supporting early referral to specialty pain care. Therefore, such a tool may act as a resource to first-line providers^4^ (eg, ED, primary care, physiotherapy). As such, the screening tool should be highly sensitive to the target condition, inexpensive, and easy to implement.^12^

PainDETECT has demonstrated reliability^6,23^ and validity^6^ in adult populations for screening NP but has not been examined in pediatric populations. The goal of this study was to provide an initial evaluation of the validity of the painDETECT in a pediatric population to inform the potential clinical and research utility of the measure.

## 2. Methods

### 2.1. Design

This was an ancillary study from a larger multisite protocol (for further details, refer to study by Heathcote et al. ^8^ and NIH Grant: R01HD083270). Regarding the validity of the painDETECT, this study specifically analyzed criterion validity and convergent validity. Criterion validity was considered the degree to which results on the painDETECT relate to a gold standard test. In the absence of a gold standard test for NP in pediatrics, our study used pain diagnosis (International Classification of Disorders - 10th Edition [ICD-10] codes^13^) rendered by a pediatric pain physician. Indicators of criterion validity were sensitivity (SE), specificity (SP), false-positive (FP), and false-negative (FN) rates. Convergent validity was considered the relationship of the painDETECT tool with other tools that measure NP (ie, quantitative sensory testing [QST]).

### 2.2. Participants

Youth with chronic pain were recruited from the Pain Treatment Service at the Boston Children's Hospital and the Pediatric Pain Management Clinic at the Stanford Children's Health when they presented for their pain evaluation. This cohort was recruited as part of a larger study examining threat learning using questionnaire and experimental methods (MRI, QST, physiological monitoring) (for further details, refer Heathcote et al.^8^ and NIH Grant: R01HD083270). Patients who were 10 to 19 years old and had pain for more than 3 months were included into the study. Exclusion criteria included significant cognitive impairment, severe medical or psychiatric disorder, pregnancy, claustrophobia, or any existing magnetic implants. The participants without chronic pain were recruited from the community and met the same inclusion/exclusion criteria with the addition of no documented current or history of chronic pain (any pain symptoms that had persisted for >3 months).

### 2.3. Study procedure

Participants completed a paper version of the painDETECT and quantitative sensory testing that assessed 2 tasks—mechanical allodynia (brush-evoked pain) and mechanical hyperalgesia (pressure pain)—as part of a battery of assessments in the larger, multisite, threat-learning study.

### 2.4. Measures

#### 2.4.1. Pain diagnosis

Pain diagnoses were collected through medical chart ICD-10 codes and cross-referenced with both patient and parent report when unclear. Participants diagnostic codes were grouped into the following pain diagnosis categories based on primary diagnoses in the medical chart: NP and non-NP diagnoses, including CRPS, diffuse musculoskeletal pain (diffuse MSK), localized musculoskeletal pain (localized MSK), and abdominal pain or pelvic pain (see Table, Supplemental Digital Content 1, available at http://links.lww.com/PR9/A209 for individual ICD-10 codes within each diagnostic category).

#### 2.4.2. PainDETECT

The painDETECT questionnaire is a self-report measure consisting of 12 items that ask youth to rate their pain intensity, characteristics of their pain such as any burning sensations, tingling or prickling, and sudden pain attacks, spread or radiation of their pain, and the course or fluctuations of their pain. The first 3 pain intensity questions are not scored because they do not inform differentiation of pain types, rather they are included for clinician reference. A total score is combined by tallying the 7 pain characteristics items for an initial score out of 35 (“*never*” = 0, “*hardly noticed*” = 1, “*slightly*” = 2, “*moderately*” = 3, “*strongly*” = 4, “*very strongly*” = 5). Points are then added or subtracted when calculating the final score, based on responses to 2 items believed to be specifically sensitive to NP: pain behavior pattern and pain radiation. Responses on these 2 items can result in points either being *added* to the patients score—3 points maximum for a max final score of 38—or *subtracted* from the patients score. Final scores are interpreted as: *negative* (0–12), which indicates that components of NP are unlikely present, *unclear* (13–18), whereby NP may be possible, and *positive* (19–38), in which NP is a probable component of their diagnosis (see Figure, Supplemental Digital Content 2, available at http://links.lww.com/PR9/A209 for painDETECT questionnaire).

#### 2.4.3. Quantitative sensory testing

Dynamic mechanical allodynia was assessed by a brush test, grazing the back of one's hand with a soft brush at a rate of approximately 3 to 5 cm/s.^11,19^ Participants were asked to report whether the brush felt “soft” or “harsh” and provide a pain score to the reported sensation between 0 and 10, where 0 is no pain and 10 is worst pain imaginable. Mechanical hyperalgesia was assessed through a pressure pain test.^3^ A pressure algometer (AlgoMed Durham, NC) was applied perpendicular to the examined point of one's nail bed. Subjects were asked to report “now” when the feeling (ie, pressure) became unpleasant or uncomfortable and then again when the feeling became painful, delineating a pain threshold and pain tolerance level in kilograms per square centimeter for each subject, respectively. Both QST stimulations were completed 3 times, and calculated averages across the 3 trials for both brush-evoked pain and pressure pain were used for analyses.

### 2.5. Statistical analysis

Descriptive statistics were calculated to describe age, gender, pain diagnoses, and painDETECT scores. To evaluate whether mean QST scores were different across painDETECT categories (positive, unclear, negative), Kruskal–Wallis tests for unequal sample sizes were conducted. In addition, to assess the strength and direction of association between QST scores and painDETECT total scores, Pearson product-moment correlation coefficients were calculated. Values were interpreted as <0.3 weak relationship, 0.3 to 0.7 moderate, and >0.7 strong.^17^

Sensitivity was considered the proportion of patients with NP that scored positively (>18) on the painDETECT and specificity as the proportion of patients with other pain conditions that scored unclear or negative (≤18) on the painDETECT. Sensitivity and specificity rates of >70% were considered acceptable. False-positive rates were calculated as the proportion of patients with other pain conditions who score positively on the painDETECT, and false-negative (FN) rates were calculated as the proportion of participants with NP who score unclear or negatively on the painDETECT.^5^

## 3. Results

### 3.1. Descriptive characteristics

One hundred sixty-five participants comprised this study cohort, of which n = 110 had chronic pain (88 females, 22 males) and n = 55 did not (39 female and 16 male participants). Participants in this study generally identified as white and female and reported living in homes with high parental educational attainment and average household incomes greater than $100,000 (detailed in Table 1). Demographic differences between participants with pain and those without pain were assessed using χ^2^ analyses to account for unequal sample sizes. Results indicated no significant difference between youth with pain and their nonpain peers across collected demographics (Table 1).

**Table 1:** Youth demographics.

Variables	Youth with pain	Nonpain peers	*P*
	N (%)	N (%)	
Age, M (SD), year	15.1 (2.33)	15.8 (3.94)	0.194
Sex			0.191
Male	22 (20.0)	16 (29.1)	
Female	88 (80.0)	39 (70.9)	
Race			0.06
White	95 (86.4)	22 (77.1)	
Black or African American	2 (1.80)	1 (2.85)	
Asian	5 (4.50)	0 (0)	
American Indian or Alaska Native	1 (0.09)	0 (0)	
Multiracial	6 (5.50)	6 (17.1)	
Choose not to answer	1 (0.90)	6 (17.1)	
Ethnicity			0.316
Not Hispanic or Latino	98 (89.9)	9 (25.7)	
Hispanic or Latino	11 (10)	21 (60.0)	
Choose not to answer	1 (0.9)		
Household income			0.839
Less than $20,000	3 (2.70)	1 (1.80)	
$20,000–$39,000	5 (4.5)	1 (1.80)	
$40,000–$59,000	10 (9.1)	3 (5.50)	
$60,000–$79,000	10 (9.1)	2 (3.60)	
$80,000–$99,000	6 (5.5)	4 (7.30)	
Greater than $100,000	69 (62.7)	28 (50.9)	
Choose not to answer	7 (6.4)	16 (29.1)	
Parental marital status			0.886
Married	84 (76.4)	33 (60.0)	
Widowed	2 (1.80)	1 (1.80)	
Divorced	7 (6.40)	3 (5.5)	
Separated	2 (1.80)	0 (0)	
Never married	8 (7.30)	2 (3.6)	
Choose not to answer	7 (6.4)	16 (29.1)	
Parental education			0.097
High school or less	10 (9.10)	0 (0)	
Vocational school/some college	15 (13.6)	4 (7.30)	
College and graduate/professional school	78 (70.9)	35 (63.6)	
Choose not to answer	7 (6.4)	16 (29.1)	
PainDETECT scores			
Total (out of 35)	11.7 (6.79)	1.42 (2.15)	0.001
Final (out of 38)	12.7 (6.76)	2.05 (2.41)	0.001
QST pain reports			
Mechanical allodynia (M (SD))	0.30 (0.64)	0.04 (0.19)	0.377
Mechanical hyperalgesia (M (SD))	21.1 (11.5)	23.2 (12.8)	0.525

Participants (N = 165) including youth with pain (n = 110) and nonpain peers (n = 55).

M, mean; N, sample; QST, quantitative sensory testing.

### 3.2. painDETECT as a screening tool

#### 3.2.1. Pain vs nonpain peers

**Figure 1.**
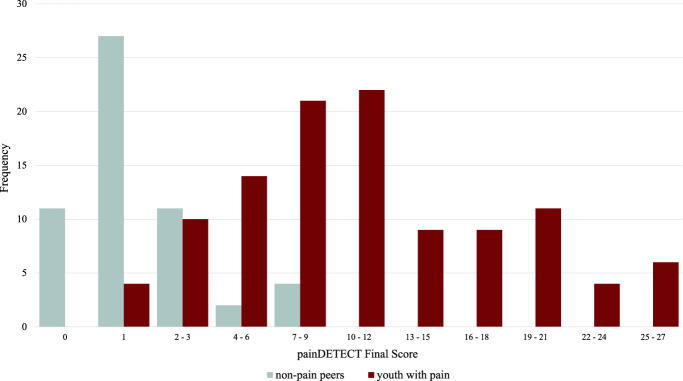
Distribution of painDETECT scores. Frequencies of painDETECT final scores for participants (N = 165) including youth with pain (n = 110) and nonpain peers (n = 55).

The painDETECT successfully differentiated youth with pain (n = 110; M_age_ = 15.1 ± 2.33 years) from those without pain (n = 55; M_age_ = 15.8 ± 3.94 years) (Fig. 1). Mean painDETECT scores for participants with pain were significantly higher than those for peers without pain (M_pain_ = 12.7 ± 6.76; M_non-pain_ = 2.05 ± 2.41; t = 11.3, *P* = <0.001). For participants with pain, final scores ranged from 1 to 27, with no final scores equal to zero and a range of scores consistent with the diversity of pain conditions reported by youth. For participants without pain, all final scores were below 10, with the majority receiving a score of zero (n = 10) or one (n = 28) as seen in Figure 1.

#### 3.2.2. Neuropathic vs other pain conditions

**Figure 2.**
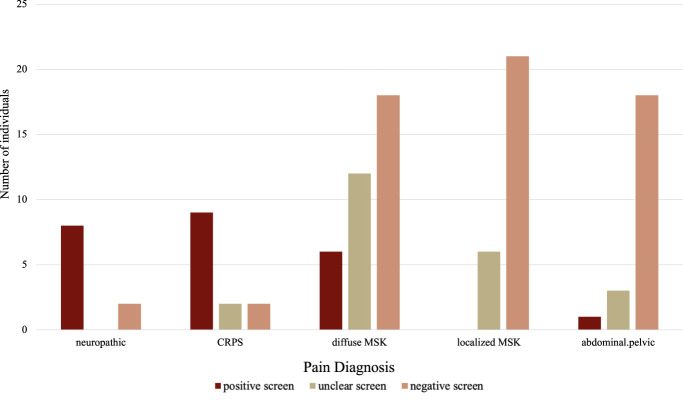
PainDETECT result by ICD-10 diagnosis. PainDETECT screening result by physician ICD-10 diagnoses grouped by diagnosis categories for youth with pain (n = 110). CRPS, complex regional pain syndrome; MSK, musculoskeletal.

Participant categorization (negative, unclear, positive) on the painDETECT stratified by diagnosis is presented in Figure 2. Among participants with a primary diagnosis of NP (n = 10; M_age_ = 13.80 ± 1.69 years), 80% were categorized as positive on the painDETECT. Among the participants diagnosed with CRPS (n = 13; M_age_ = 14.69 ± 2.32 years), 69% were categorized as positive by painDETECT. By contrast, among participants diagnosed with localized MSK pain (n = 27; M_age_ = 14.78 ± 2.39 years), 0% were categorized as positive by painDETECT, whereas among participants with diffuse MSK pain (n = 38; M_age_ = 15.63 ± 2.11 years), 21% were categorized as positive, and of those with abdominopelvic pain (n = 22; M_age_ = 15.32 ± 2.75 years), 5% scored positively on the painDETECT. Two participants were excluded from diagnostic categorization analyses because they had no clear ICD-10 diagnoses.

#### 3.2.3. Sensitivity and specificity

The painDETECT demonstrated acceptable SE (80%) but poor SP (33%). The FN rate was 20%, and the FP rate was 77%. When the reference test was redefined to consider CRPS as a type of NP, at a cutoff score of >18, the SE was acceptable (74%), and the SP was much improved (71%). Table 2 provide further details on SE, SP, FP, and FN rates across analyses.

**Table 2:** Indicators of criterion validity.

Interpretation of painDETECT and pain diagnoses	Indicators of validity			
	Sensitivity	Specificity	False negative	False positive
Cutoff score >18				
NP only	80	33	20	77
NP and CRPS	74	71	26	29

CRPS, complex regional pain syndrome; NP, neuropathic pain.

### 3.3. Quantitative sensory testing and painDETECT categories

**Figure 3.**
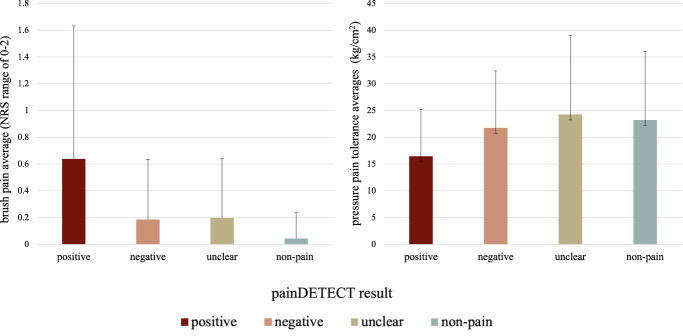
QST pain report by painDETECT result. Mean mechanical allodynia and mechanical hyperalgesia QST pain reports by painDETECT screening results for participants (N = 165). Positive screen is defined as painDETECT score ≥19 and suggests that a neuropathic pain component is likely (>90%). Unclear screen defined as painDETECT score between 13 and 18 and suggests an ambiguous result and suggests that a neuropathic pain component can be present. Negative screen defined as painDETECT score ≤12 and suggests that a neuropathic pain component is unlikely (<15%). NRS, Numerical Rating Scale; QST, quantitative sensory testing.

Average pain reports for the brush pain QST, measuring mechanical allodynia, aligned with painDETECT results. That is, participants who scored positively on the painDETECT reported higher brush-evoked pain, on average, than their peers (Fig. 3). Average pain tolerance reported during pressure pain QST, measuring mechanical hyperalgesia, did not differ across painDETECT groups.

#### 3.3.1. Mechanical allodynia

Results of the Kruskal–Wallis H test revealed a statistically significant difference in brush-evoked pain ratings between the painDETECT scores (H(2) = 8.33, *P* = 0.016). Post hoc Dunn test showed higher pain ratings in response to brush sensory testing for participants in the positive screen group compared with those in the negative (*P* = 0.006) and nonpain peer (*P* = <0.001) groups (Table 3). Pearson correlation coefficients also indicated a significant positive relationship between brush-evoked pain and painDETECT total score (*r*(108) = 0.248, *P* = 0.009) such that as painDETECT scores increased (ie, more NP features present), brush-evoked pain ratings also increased.

**Table 3:** Krusil–Wallis results for brush pain and pressure pain.

PainDETECT results	N	Brush pain	Pressure pain	Mean rank	
		Mean (SD)	Mean (SD)	Brush pain	Pressure pain
Positive	26	0.640 (0.994)	16.46 (8.77)	105.7	59.5
Negative	61	0.186 (0.449)	21.73 (10.64)	81.8*	84.1
Unclear	23	0.196 (0.446)	24.25 (14.8)	84.4	86.7
Nonpain	54	0.043 (0.195)	23.2 (12.85)	71.4*	86.6

Higher scores on Brush test refer to more mechanical allodynia (ie, higher exacerbated response to innocuous pain). Lower scores on pressure test refer to more mechanical hyperalgesia (ie, higher exacerbated response to pain stimuli).

* *P*-value <0.001 compared with positive result.

#### 3.3.2. Mechanical hyperalgesia

Results of the Kruskal–Wallis H test for pressure pain did not reveal a significant difference across groups (H(2) = 5.32, *P* = 0.07). The mean pain tolerance score was lowest within the positive screen group (M = 16.46 ± 8.77) followed by the negative screen group (M = 21.73 ± 10.64), and the unclear screen group had the highest mean pain tolerance score (M = 24.25 ± 14.8) (Table 3). In addition, Pearson correlation coefficient revealed a weak nonsignificant relationship between total painDETECT score and mean pressure pain tolerance score (*r*(106) = −0.1, *P* = 0.303).

## 4. Discussion

This study is one of the first to assess the validity of an established adult neuropathic pain screening tool in a pediatric chronic pain population. Overall, preliminary data from this study suggest that the painDETECT has the potential as a screening tool for NP among pediatric patients. That is, painDETECT demonstrated good sensitivity to physician ICD-10 diagnoses such that when the patient was diagnosed with neuropathic pain, they often screened positively on painDETECT. Study results do not suggest painDETECT to be a valid screening tool to discriminate among pain diagnoses broadly but holds potential as a brief, easy-to-implement, and inexpensive front-line screening tool for pediatric patients presenting with primary pain conditions to identify when features of NP are present and should be evaluated further.

In this study, sensitivity was acceptable (80%), but specificity was quite poor (33%), in contrast to previous validation studies within adult populations, which indicated excellent SE (84%) and SP (84%).^6^ Our analyses indicate a significant contributor to poor SP, and a high-FP rate in the large proportion of participants with CRPS who received positive screens on the painDETECT. Therefore, there is a need to refine or develop a screening tool that can effectively delineate between patients with neuropathic pain and those with CRPS. Of note, although the initial validation of the painDETECT was conducted across multiple pain centers, inclusion criteria restricted participation to those with low back pain.^6^ Not surprisingly, because CRPS primarily presents in the peripheral limbs, the initial validation study did not include any participants with CRPS and thus did not face the same challenges observed in this study regarding specificity. In addition, this tool has not been tailored to consider the unique developmental needs of pediatric patients, which may also be contributory to the poor SP rate.

Despite these challenges to SP, the SE rate is similar to previous validation studies within an adult population.^6^ That is, of those diagnosed by physicians as having NP, a high percentage (80%) received a final score greater than 18 and a positive NP screen, indicating that painDETECT shows initial promise in matching physician diagnosis. Moreover, the high SE rate further supports the notion that screening tools can be a useful tool in nonpain specialty clinics and ultimately facilitate referral to a tertiary pain clinic and thus a diagnosis and initiation of appropriate treatment.

Regarding convergent validity, our QST results are equivocal both aligning and diverging with current literature that highlights mechanical allodynia and hyperalgesia as key features of NP.^1,2^ To this end, although both mechanical allodynia and hyperalgesia were observed in participants with a positive painDETECT screen, significant differences between the groups only existed for brush-evoked pain. That is, mean brush-evoked pain scores were significantly higher among those who screened positive on painDETECT, and painDETECT scores were significantly positively related to brush-evoked pain scores such that as painDETECT scores increased (ie, more indicators of NP) so did average pain ratings in response to the QST brush test, consistent with mechanical allodynia.^2,11,19^ Regarding mechanical hyperalgesia, although mean pain tolerance scores were lowest among those who scored positive on painDETECT, consistent with the existing literature, results of the correlation analysis between painDETECT scores and QST pressure pain tolerance scores were not significant.^1^

Taken together, our results indicate that painDETECT screening tool can provide clinicians useful information for directing care and prompting appropriate follow-up. Because painDETECT is a screening tool and not meant to be diagnostic in nature, results or categorizations should be used to direct further assessment or evaluation and inform referral recommendations^12,15,16,21^ and should not be used to render a diagnosis. For patients who screen positively on painDETECT, urgent follow-up with a physician with pediatric pain expertise is recommended to confirm a diagnosis and initiate appropriate treatments. Clinicians who use this tool in settings with patients with CRPS should take caution in interpreting a positive test result. PainDETECT instructions indicate that a score >18 indicates that a “NP component is likely”; however, given our high FP rate, clinicians should be aware that a positive result may also be seen in patients with CRPS.

Most importantly, youth with undiagnosed NP require a streamlined path to treatment.^9,27^ Therefore, a tool that is more sensitive over specific, is preferred as differentiating NP from other types of pain and flagging NP symptomology,^12,14,26^ is vital for the appropriate referral to a pain clinic wherein NP can be diagnosed and treatment can be initiated.^27^ Nevertheless, specificity of the tool needs to be improved upon so that we do not misuse resources because of the possible excessive amount of false-positive results. At present, painDETECT warrants continued examination and refinement. That is, despite some promising SE rates, painDETECT also demonstrated low specificity in our study and has been questioned previously when used in generalized pain populations.^24^

### 4.1. Limitations

The results of this study offer support for the continued evaluation of the painDETECT and use of screening tools within pediatric populations. However, the limitations of this study warrant attention and prompt ongoing research. (1) *Small sample size and retrospective design.* One limitation of this study is the small sample size of youth with NP (N = 10). This sample may not represent the diversity of patients with NP, across ages, sexes, neurological lesions, and diseases, thus generalizability is a limitation. Future studies with larger sample sizes are needed to ensure adequate power to detect differences and may necessitate harnessing multicenter collaborations. In addition, this study was retrospective as patients who completed the painDETECT already had received a diagnosis from a tertiary pain clinic. Moreover, in the absence of a gold standard diagnostic test for neuropathic pain (in children or adults), all studies of criterion validity are subject to using a reference test in the absence of a gold standard test. Therefore, the reader must interpret the validity of the painDETECT in relation to physician diagnosis and not the *absolute truth* as is the case with a gold standard test. (2) *Lower specificity.* Sensitivity was acceptable (80%), but SP was quite poor (33%) and FP-rate was high (77%). The painDETECT was intended for use with adults with lumbar radiculopathy, a type of peripheral NP. Using this tool in pediatric setting with diverse types of NP (central and peripheral) warrants additional research, including content validity testing. Low specificity and high FP rate were particularly true for patients with diffuse MSK pain and CRPS. Among youth with diffuse MSK, the painDETECT demonstrated the highest variability in patient categorization. Although this may be a function of the broad diagnostic category of diffuse MSK pain, it does prompt further evaluation of the tool's utility in patients with more variable pain presentations.^7^ (3) *Predictive strength dependent on population.* It is possible that the painDETECT is strong in adult and homogenous pain populations, whereas other pediatric screening tools for NP may be preferrable among pediatric patients with diverse pain presentations. To this end, if using the painDETECT in a pediatric population with diverse pain problems, particularly one that includes CRPS, we recommend caution in the interpretation of screening results given the limitations noted above. By contrast, if employing the painDETECT in a population where CRPS is highly unlikely (eg, in cancer patients receiving chemotherapy), the painDETECT could hold even more potential.

### 4.2. Future directions

*Future work would benefit from* (1) *prospective design and painDETECT's effectiveness.* Future studies should employ a prospective study design, administering the tool before the patient receives their diagnosis from a physician, with physicians blinded to the results of the painDETECT. Such design will assess for superiority and accuracy of the tool in accurately categorizing pain symptoms. Future studies should also evaluate whether a positive neuropathic screen streamlines referral practices, reduces clinic visit wait times, increases access to appropriate integrated treatments, and decreases diagnostic uncertainty for patients, families, and providers. (2) *Validation studies.* The design purpose of the painDETECT was for adults with low back pain, and future research is needed to determine the content validity of the painDETECT in applying this to diverse pain diagnoses and in a pediatric population. Content validity should be examined by employing cognitive interviews to assess pediatric patients' interpretation of the measure items and affirm whether the meaning of the painDETECT questions is translating as expected. It is possible that this research may result in altered question formatting, scoring, or language that is more sensitive and developmentally appropriate for pediatric patients, which should theoretically improve the specificity and sensitivity of the measure. (3) *Deploy in various pain populations*. Further research is needed that deploys the painDETECT in diverse pain populations (eg, cancer-related NP, post-surgical NP). To this end, our results suggest that if one is using the painDETECT in a pediatric population with diverse pain problems, particularly one that includes CRPS, we recommend caution in the interpretation of screening results given the limitations noted above. By contrast, if employing the painDETECT in a population where CRPS is highly unlikely (eg, in cancer patients receiving chemotherapy), the painDETECT could hold even more potential. In addition, given the diverse nature of pain diagnoses in pediatric pain clinics, including CRPS, the need for a tool that can screen for NP and accurately distinguish CRPS from NP is clear. The Pediatric PainSCAN is a tool that is under development to address this need and was developed specifically for pediatric populations with NP and CRPS, without using the adult tool developed for radiculopathies.^12^

### 4.3. Conclusions

These results support painDETECT to be sensitive to NP symptom presentation, highlighting the advantages of using a screening tool in pediatric pain populations. Results also prompt the continued exploration of this tool and its features in pediatric populations with diverse pain problems. This article further emphasizes the need for continued efforts to develop and implement highly sensitive screening tools into clinical practice within pediatric populations so as to expedite appropriate referrals and initiate identification of NP to ameliorate diagnostic delays and ultimately improve treatment outcomes.

## Disclosures

The authors have no conflict of interest to declare.

## Appendix A. Supplemental digital content

Supplemental digital content associated with this article can be found online at http://links.lww.com/PR9/A209.
