# Supplementary material for: Application of PainDETECT in pediatric chronic pain: how well does it identify neuropathic pain and its characteristics?
Source: Pain Rep. 2023 Nov 28;8(6):e1109. doi: 10.1097/PR9.0000000000001109 (PMC10686590; doi:10.1097/PR9.0000000000001109)
Supplement: Supplementary file 1 [file painreports-8-e1109-s001.pdf]

Table, Supplemental Digital Content 1. Categorization of physician ICD-10 diagnoses and ICD-10 codes

Diagnostic Category	ICD-10 Diagnosis	ICD-10 Code
**Neuropathic** (n=10)		
	Genitofemoral neuralgia	G57.22
	Neuralgia and neuritis	M79.2
	Peroneal Neuropathy	S84.1
	Right ulnar neuropathy	G56.21
	Sensory neuropathy	G60.0
**CRPS** (n=13)		
	Complex regional pain syndrome	G90.5
**Diffuse MSK** (n=38)		
	Arthritis	M13.80
	Chronic pain, generalized	G89.2
	Diffuse amplified pain syndrome	M79.19
	Ehlers-Danos syndrome	Q79.6
	Fibromyalgia	M79.7
	Hypermobility syndrome	M35.7
	Myofascial pain syndrome	M79.18
	Post viral syndrome	G93.3
	Patellaformal pain syndrome	M22.2
	Tendinitis	M65.2
**Localized MSK** (n=27)		
	Chronic pain in joint	M25.5
	Chronic pain in localized limb	M79.60
	Intercostal pain	R07.82
**Abdominal & Pelvic** (n=22)		
	Celiac	K90.0
	Endometriosis	N80.9
	Generalized abdominal pain	R10.84
	Irritable Bowel Syndrome	K58
	Pelvic pain	R10.2

Figure, Supplemental Digital Content 2. painDETECT questionnaire

painDETECT PAIN QUESTIONNAIRE

Date: ____________ **Patient:** **Last name:** ______________ **First name:** ______________

How would you assess your pain **now**, at this moment?

0 1 2 3 4 5 6 7 8 9 10

none — max.

How strong was the **strongest** pain during the past 4 weeks?

0 1 2 3 4 5 6 7 8 9 10

none — max.

How strong was the pain during the past 4 weeks **on average**?

0 1 2 3 4 5 6 7 8 9 10

none — max.

Mark the picture that best describes the course of your pain:

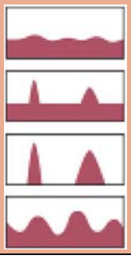

- **Persistent pain with slight fluctuations** ☐
- **Persistent pain with pain attacks** ☐
- **Pain attacks without pain between them** ☐
- **Pain attacks with pain between them** ☐

Please mark your **main area of pain**

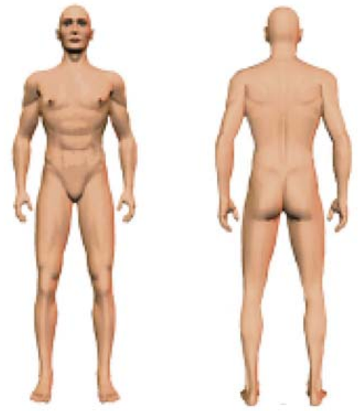

Does your pain radiate to other regions of your body? yes ☐ no ☐

If yes, please draw the direction in which the pain radiates.

Do you suffer from a burning sensation (e.g., stinging nettles) in the marked areas?

never ☐ hardly noticed ☐ slightly ☐ moderately ☐ strongly ☐ very strongly ☐

Do you have a tingling or prickling sensation in the area of your pain (like crawling ants or electrical tingling)?

never ☐ hardly noticed ☐ slightly ☐ moderately ☐ strongly ☐ very strongly ☐

Is light touching (clothing, a blanket) in this area painful?

never ☐ hardly noticed ☐ slightly ☐ moderately ☐ strongly ☐ very strongly ☐

Do you have sudden pain attacks in the area of your pain, like electric shocks?

never ☐ hardly noticed ☐ slightly ☐ moderately ☐ strongly ☐ very strongly ☐

Is cold or heat (bath water) in this area occasionally painful?

never ☐ hardly noticed ☐ slightly ☐ moderately ☐ strongly ☐ very strongly ☐

Do you suffer from a sensation of numbness in the areas that you marked?

never ☐ hardly noticed ☐ slightly ☐ moderately ☐ strongly ☐ very strongly ☐

Does slight pressure in this area, e.g., with a finger, trigger pain?

never ☐ hardly noticed ☐ slightly ☐ moderately ☐ strongly ☐ very strongly ☐

(To be filled out by the physician)

never	hardly noticed	slightly	moderately	strongly	very strongly
☐ x 0 = 0	☐ x 1 = ☐	☐ x 2 = ☐☐	☐ x 3 = ☐☐	☐ x 4 = ☐☐	☐ x 5 = ☐☐

Total score ☐☐ **out of 35**

Development/Reference: R. Freynhagen, R. Baron, U. Gockel, T.R. Tölle / Curr Med Res Opin, Vol.22, No. 10 (2006) ©2005 Pfizer Pharma GmbH
painDETECT questionnaire, ©2005 Pfizer Pharma GmbH, used with permission.

painDETECT

SCORING OF PAIN QUESTIONNAIRE

Date: ____________ Patient: Last name: ______________ First name: ______________

Please transfer the total score from the pain questionnaire:

Total score ☐☐

Please add up the following numbers, depending on the marked pain behavior pattern and the pain radiation. Then total up the final score:

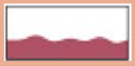	Persistent pain with slight fluctuations	0	
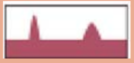	Persistent pain with pain attacks	- 1	if marked, or
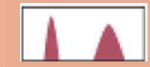	Pain attacks without pain between them	+ 1	if marked, or
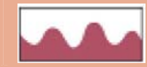	Pain attacks with pain between them	+ 1	if marked
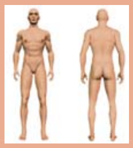	Radiating pains?	+ 2	if yes
	**Final score**	☐☐	

Screening Result

Final score

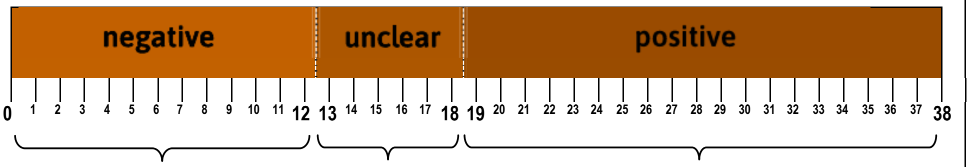

A neuropathic pain component is unlikely (< 15%)	Result is ambiguous, however a neuropathic pain component can be present	A neuropathic pain component is likely (> 90%)

This sheet does not replace medical diagnostics.
It is used for screening the presence of a neuropathic pain component.

Development/Reference: R. Freynhagen, R. Baron, U. Gockel, T.R. Tölle / Curr Med Res Opin, Vol.22, No. 10 (2006)

©2005 Pfizer Pharma GmbH
